# Advances and Emerging Techniques in Y-90 Radioembolization for Hepatocellular Carcinoma

**DOI:** 10.3390/cancers17091494

**Published:** 2025-04-29

**Authors:** Elliott L. Fite, Mina S. Makary

**Affiliations:** 1 College of Medicine, The Ohio State University, Columbus, OH 43210, USA; elliott.fite@osumc.edu; 2 Department of Radiology, The Ohio State University Medical Center, Columbus, OH 43210, USA

**Keywords:** hepatocellular carcinoma, transarterial radioembolization, Yttrium-90

## Abstract

Hepatocellular carcinoma (HCC) is the fourth leading cause of cancer deaths worldwide. Despite the high incidence of HCC, mortality remains high, with an estimated 5-year survival rate of less than 20%. Surgical resection represents a potential curative treatment for HCC; however, less than 20% of patients with HCC are candidates for surgical resection. In patients with unresectable HCC, Yttrium-90 (Y90) transarterial radioembolization (TARE) has emerged as an innovative treatment option. This locoregional therapy delivers high doses of radiation directly to liver tumors via intra-arterial injection, allowing for the targeted destruction of malignant cells while sparing surrounding healthy tissue. In this review, we will explore the latest advances in Y90 TARE for the treatment of HCC, focusing on key developments such as the following: (1) improvements in radiation lobectomy and segmentectomy techniques, (2) the introduction of personalized dosimetry, (3) the integration of combination therapies, (4) the use of imageable microspheres, (5) pressure-enabled Y90 delivery systems, and (6) the application of Y90 surrogates.

## Simple Summary

Yttrium-90 (Y90) transarterial radioembolization (TARE) is a locoregional treatment for hepatocellular carcinoma (HCC) that delivers targeted radiation through microspheres injected into the hepatic artery, effectively controlling tumors while minimizing damage to healthy liver tissue. Advances in imaging, dosimetry, and techniques such as radiation segmentectomy and lobectomy have further enhanced its precision and effectiveness. Combining TARE with immunotherapy has additionally demonstrated promising results, increasing tumor response rates and survival. Additionally, innovations such as imageable microspheres, pressure-enabled delivery systems, and alternative radionuclides such as Holmium-166 are improving the treatment accuracy and expanding therapeutic options. As research progresses, TARE continues to evolve as a key component in HCC management, offering personalized, effective, and minimally invasive treatment solutions.

## 1. Introduction

Yttrium-90 (Y90) radioembolization, clinically known as transarterial radioembolization (TARE), is a paradigm-shifting treatment modality for both primary and secondary liver malignancies. This innovative approach integrates selective arterial embolization with the precise application of beta-emitting radiotherapy, leveraging the physical properties of Y90 to deliver highly localized, tumoricidal radiation doses while minimizing collateral damage to healthy hepatic parenchyma [1]. The minimally invasive nature of TARE underscores its versatility, offering an effective and safer alternative to traditional systemic and surgical interventions for hepatic tumors [2,3].

Despite the widespread use of systemic therapies such as sorafenib, lenvatinib, and immune checkpoint inhibitors, the overall prognosis for advanced hepatocellular carcinoma (HCC) remains poor, with limited progression-free survival and modest response rates. Surgical resection and liver transplantation are curative options but are often restricted by underlying liver dysfunction or the extent of the tumor. Transarterial chemoembolization (TACE) [4], while effective in intermediate-stage HCC, is associated with heterogeneous response rates and the risk of post-embolization syndrome [5,6,7]. These limitations underscore the need for more targeted, durable, and liver-sparing therapies, thereby justifying the continued development and refinement of Y90 TARE.

The efficacy of TARE is exemplified by its capacity to achieve robust tumor control, significantly extend survival outcomes, and enhance patients’ quality of life, making it a cornerstone in the management of HCC and metastatic liver disease [8]. Moreover, the advent of advanced imaging and dosimetry techniques has elevated the precision of TARE, enabling highly individualized therapeutic strategies [1,2,9,10].

This review provides a detailed examination of TARE, encompassing its sophisticated methodologies, comprehensive patient selection criteria, and emergent innovations, such as imageable microspheres and novel radionuclide surrogates such as Holmium-166 (Ho-166) [11]. The articles included in this review were identified using the Pubmed database, with specific searches for each topic discussed in this review (i.e., personalized dosimetry, combinations therapies with TARE, and imageable microspheres, etc.). By synthesizing findings from pivotal clinical trials and exploring future directions, this article offers a thorough assessment of the evolving role of TARE in contemporary oncological practice, reaffirming its status as a vital component of personalized cancer therapy.

## 2. Overview of Transarterial Radioembolization

### 2.1. TARE Technique

TARE employs the targeted delivery of microspheres loaded with Y90, a beta-emitting radionuclide, through the hepatic artery to selectively irradiate hepatic tumors [12,13]. The procedure commences with a detailed planning angiography to map the hepatic vasculature and identify non-target vessels [14]. To prevent inadvertent radiation delivery to unintended areas, the pre-treatment embolization of extrahepatic arteries, such as the gastroduodenal artery, can be performed [15].

Dosimetry planning is paramount and involves sophisticated calculations to determine the optimal activity of Y90, ensuring maximum tumor irradiation while safeguarding surrounding normal liver parenchyma [3]. These calculations incorporate factors such as tumor volume, liver volume, lung shunt fraction, and perfused liver segment, typically using partition model or voxel-based dosimetry approaches [16]. In the partition model, activity is calculated using the Medical Internal Radiation Dose (MIRD) schema and accounts for the differential uptake in tumor versus normal liver to deliver a therapeutic tumor dose (commonly > 120 Gy), while minimizing the exposure to healthy tissue and lungs [17]. Voxel-based dosimetry, enabled by advanced imaging such as 99 mTc-MAA SPECT/CT and Y90 PET/CT, provides three-dimensional dose maps that improve the spatial accuracy and allow for further refinement of the planning of treatment [10,17].

Potential risks of non-target radiation exposure remain a significant concern. An inadequate dosimetric assessment or technical limitations can lead to the unintended irradiation of healthy liver tissue, gastrointestinal structures, or lungs, potentially resulting in radiation-induced liver disease (RILD), gastric ulceration, or radiation pneumonitis [18]. Advances in imaging and computational modeling have mitigated these risks by enabling a more precise localization of microsphere deposition and more reliable dose estimates. Following microsphere administration, post-procedural imaging, including SPECT or PET, is conducted to verify an accurate microsphere distribution and assess its therapeutic efficacy [8].

### 2.2. Outcomes in TARE Therapy

Robust clinical evidence has highlighted the efficacy of Y90 TARE in achieving tumor control and extending survival. A comprehensive 15-year study involving 1000 HCC patients treated with TARE demonstrated promising survival outcomes [19]. In this cohort, patients with Child–Pugh A cirrhosis and BCLC stage A disease achieved a median overall survival (OS) of 47.3 months [19]. Those with BCLC stage B and C had a median OS of 25.0 and 15.0 months, respectively [19]. These findings underscore the efficacy of TARE across various stages of HCC, supporting its adoption as a first-line locoregional therapy in appropriate patient populations.

### 2.3. Patient Selection

Appropriate patient selection is paramount to optimizing the therapeutic efficacy and safety of Y90 TARE. Ideal candidates for this intervention include patients with unresectable HCC or liver-dominant metastatic disease who demonstrate adequate hepatic function and performance status. Selection criteria encompass several key aspects, which are outlined in Table 1.

Candidates should have a preserved hepatic function, typically classified as Child–Pugh class A or B (score ≤ 7), indicating minimal cirrhosis-related impairment (Table 1) [21]. Patients with decompensated cirrhosis (Child–Pugh class C) or severe liver dysfunction are generally not considered suitable due to the high risk of liver failure [1,21,22]. TARE is most effective in patients with liver-dominant HCC and limited extrahepatic disease [21]. Ideal candidates have multifocal but liver-confined disease, with minimal or no extrahepatic metastasis [21]. The absence of significant biliary obstruction is also important, as this can increase the risk of post-TARE complications.

Favorable hepatic arterial anatomy is essential for the safe and effective delivery of Y90 microspheres. The absence of significant arteriovenous (AV) shunting is crucial, as AV shunts can lead to unintended radiation exposure to the lungs, increasing the risk of radiation pneumonitis (Table 1) [23]. While TARE can be used in patients with segmental or lobar portal vein thrombosis (PVT) [22], those with main portal vein occlusion require careful assessment to avoid exacerbating hepatic ischemia [22,23]. Patient performance status, as measured by the Eastern Cooperative Oncology Group (ECOG) scale, is a predictor of the treatment’s tolerance and survival. Candidates for TARE should have an ECOG performance status of 0–2, indicating full activity or mild limitations in daily activities (Table 1) [1,21]. Patients with ECOG ≥ 3 are generally poor candidates, as they are less likely to tolerate treatment and may have a poor prognosis [21].

Certain conditions preclude the safe use of TARE, including uncorrectable arteriovenous or portosystemic shunting, substantial pulmonary shunting, severe renal or hepatic insufficiency, extensive extrahepatic disease, and poor baseline liver function (Child–Pugh C) (Table 1) [1,12,21]. These factors increase the risk of complications and diminish the potential benefits of the procedure. In summary, TARE is a well-established locoregional therapy for unresectable HCC, particularly in patients with preserved liver function, liver-confined disease, and adequate performance status. A careful evaluation of liver function, tumor burden, vascular anatomy, performance status, and contraindications ensures the appropriate patient selection, maximizing the therapeutic benefits while minimizing risks.

### 2.4. Safety of TARE

The safety profile of TARE is highly favorable, with most complications being mild and transient. Common side effects, such as post-embolization syndrome—characterized by fatigue, nausea, and abdominal discomfort—typically resolve within days. Rare but severe adverse events include radiation-induced liver disease (RILD) and gastrointestinal ulceration resulting from non-target embolization. RILD is influenced by dosing factors such as a high mean absorbed radiation dose to non-tumorous liver tissue, whole-liver treatment, and inadequate dose partitioning, especially in patients with underlying cirrhosis or a poor baseline liver function, as indicated by lower serum albumin and higher bilirubin levels [24]. Rigorous patient selection and advanced imaging techniques have proven essential in mitigating these risks [3,6].

Clinical trials provide further validation of TARE’s safety in HCC patients. The SARAH trial, comparing TARE to sorafenib in advanced HCC, demonstrated significantly lower rates of grade 3 or higher adverse events in the TARE group (40% versus 63%) [25]. Specifically, the most frequent grade 3 or worse treatment-related adverse events in the TARE group included fatigue (9%), liver dysfunction (11%), increased laboratory liver values (9%), and hematological abnormalities (10%). In contrast, the sorafenib group experienced higher incidences of fatigue (19%), liver dysfunction (13%), increased liver values (7%), hematological abnormalities (14%), and notably higher rates of diarrhea (14% vs. 1%) and hand–foot skin reaction (6% vs. <1%) [25]. Median OS for the TARE group was 8.0 months (95% CI, 6.7–9.9) versus 9.9 months (CI, 8.7–11.4) in the sorafenib group [25]. Given the similar effects on OS between TARE and sorafenib, the favorable safety profile of TARE makes it an appealing treatment option in HCC care.

Similarly, the SIRveNIB trial, which also compared TARE with sorafenib, reported that fewer patients in the TARE group experienced serious adverse events (20.8% vs. 35.2%), and the mean duration of side effects was shorter in the TARE arm [10].

These findings emphasize the necessity of meticulous patient selection and adherence to procedural protocols to ensure optimal therapeutic outcomes with minimal risks. The lower incidence of severe adverse events and the shorter duration of side effects associated with TARE highlight its favorable safety profile compared to sorafenib in the treatment of advanced HCC.

## 3. Radiation Segmentectomy and Lobectomy Techniques

Radiation segmentectomy and lobectomy represent highly selective and sophisticated applications of TARE, wherein high-dose radiation is delivered to specific liver segments or lobes. These approaches differ significantly in their therapeutic intent and scope: segmentectomy targets small, localized regions for precise tumor ablation, making it particularly suitable for early-stage HCC [9], while lobectomy addresses larger, more diffuse tumors, often as a preparatory measure for future curative interventions [26]. This differentiation is foundational to understanding the unique applications of these techniques in hepatic malignancy management, as they strive to achieve complete tumor ablation while preserving maximal healthy liver tissue.

### 3.1. Radiation Segmentectomy

**Figure 1:**
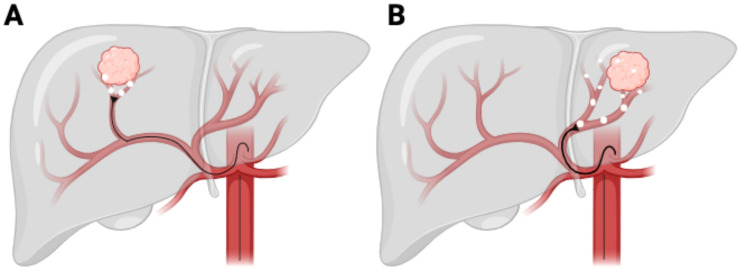
Y90 TACE procedure. (**A**) Radiation segmentectomy. (**B**) Radiation lobectomy.

Radiation segmentectomy involves the focused delivery of high-dose radiation to a single liver segment or sub-segment (Figure 1A) [27]. This approach is predominantly utilized in patients with small, localized tumors, particularly those in early-stage HCC [28]. By concentrating the radiation dose within a limited area, segmentectomy seeks to achieve complete tumor ablation while conserving the majority of the liver’s functional parenchyma. This makes it a viable alternative to surgical resection, especially for patients deemed ineligible for surgery due to comorbidities or anatomical constraints, such as the tumor’s proximity to major hepatic vessels, portal vein thrombosis, or insufficient liver remnant volume post-treatment [27,28].

Technical considerations are crucial in the successful implementation of radiation segmentectomy for HCC. Patient selection is guided by tumor size, vascular anatomy, and underlying liver function, ensuring optimal therapeutic outcomes. Clinical studies have shown that patients with tumors smaller than 5 cm and a well-preserved liver function (Child–Pugh A or well-compensated B) achieve superior outcomes [29]. The use of advanced imaging modalities such as contrast-enhanced MRI and PET-CT assists in precisely delineating the tumor burden and vascular supply, which is essential for accurate radiation planning.

Dosimetric analysis is vital for optimizing radiation segmentectomy. Studies have demonstrated that achieving an absorbed dose of >190 Gy to the tumor correlates with improved response rates, while maintaining a non-tumoral liver exposure below hepatotoxic thresholds (<70 Gy) reduces the risk of liver dysfunction [16]. Personalized dosimetry approaches using SPECT/CT-based Y90 distribution mapping have resulted in significantly higher tumor control rates [29]. Moreover, catheter positioning and microcatheter techniques significantly influence the radiation delivery’s efficiency, impacting local control and minimizing non-target embolization [9]. These technical parameters underscore the importance of individualized treatment planning in maximizing the therapeutic benefits while preserving hepatic function.

The LEGACY trial evaluated the therapeutic efficacy and safety of high-dose (400 Gy) radiation segmentectomy in patients diagnosed with early- to intermediate-stage HCC. Conducted as a prospective, multicenter study involving 162 participants, the trial yielded an objective response rate (ORR) of 90.6% [30], underscoring Y90 TARE’s capability as a highly efficacious intervention for unresectable HCC. Furthermore, in patients meeting the Milan criteria for liver transplantation, the study reported a median OS of 44.6 months [30], substantiating the modality’s potential to serve as an effective bridging therapy toward transplantation.

Beyond its efficacy, Y90 TARE exhibited a favorable safety profile characterized by a low incidence of severe adverse events, with grade 3 or higher toxicities reported in only 4.2% of patients [30]. These results highlight the dual advantages of this treatment: achieving robust tumor control while preserving the patient’s quality of life and minimizing toxicity.

The findings from the LEGACY trial significantly augment the existing body of evidence supporting Y90 TARE as a pivotal therapeutic option for patients ineligible for surgical resection or immediate transplantation. The demonstrated capacity of Y90 TARE to achieve durable local control and enhance survival outcomes reaffirms its integral role in the multidisciplinary management paradigm of HCC.

### 3.2. Radiation Lobectomy

Radiation lobectomy, in contrast, involves the administration of a high radiation dose to an entire hepatic lobe (Figure 1B), primarily for patients with a more extensive tumor burden, often in an advanced-stage HCC setting [31]. This approach serves the dual function of achieving tumor control while simultaneously inducing contralateral liver hypertrophy, a key consideration in patients who may subsequently undergo surgical resection or transplantation [32].

Radiation lobectomy is most effective for patients with unilobar disease or a dominant tumor burden confined to one lobe, as studies have demonstrated that restricting radiation to a single lobe can induce hypertrophy of the contralateral liver and improve post-treatment outcomes [33]. The tumor’s proximity to major hepatic vessels and bile ducts must also be considered to prevent excessive radiation exposure to critical structures [34]. The liver’s capacity to sustain function post-treatment is critical, and evaluation is performed using the Child–Pugh classification, Model for End-Stage Liver Disease (MELD) score, and indocyanine green retention tests [35]. Patients with well-compensated liver disease (Child–Pugh A or well-compensated B) are the best candidates, as those with compromised hepatic function face a higher risk of liver failure after radiation exposure [35,36]. Additionally, the hepatic arterial and venous anatomy dictate the feasibility of selective catheterization for optimal radiation delivery. The presence of portal vein thrombosis, arteriovenous shunting, or aberrant vascular structures requires a precise catheter positioning and dose modulation to optimize the radiation distribution while minimizing toxicity [25].

Dosimetry optimization remains central to radiation lobectomy. An absorbed dose of ≥120 Gy to the treated lobe yields optimal tumoricidal effects, while limiting exposure to the untreated hepatic segments below 50 Gy reduces the hepatotoxicity risk [16]. Selective catheterization via segmental hepatic arteries ensures a maximal radiation delivery to the targeted region while reducing unintended embolization [9]. Post-treatment verification through 90Y PET/CT imaging is essential in confirming dose deposition and evaluating the treatment’s efficacy.

A critical advantage of radiation lobectomy is its ability to induce substantial hypertrophy of the contralateral lobe within 4–6 weeks, thereby broadening the eligibility of patients for definitive surgical intervention [33]. Chow et al. show that approximately 85% of patients exhibited significant hypertrophy of the untreated lobe, with a median volumetric increase of 40% [33]. This hypertrophy was sufficient to enable subsequent resection in nearly 70% of patients who initially did not meet surgical criteria [33]. Furthermore, the study reported a correlation between the hypertrophy response and improved overall survival, reinforcing the role of radiation lobectomy as a bridge to curative intervention [33]. These findings underscore the potential of radiation lobectomy as a vital tool in the multimodal management of HCC, expanding treatment options and improving long-term outcomes for patients who would otherwise have limited therapeutic choices.

### 3.3. Comparative Considerations and Clinical Implications

Although both radiation segmentectomy and lobectomy are tailored TARE strategies, their indications are distinct yet complementary. Segmentectomy is most effective for early-stage, localized HCC, providing a high likelihood of curative intent without significantly compromising hepatic function. Conversely, lobectomy serves as a strategic downstaging modality for advanced-stage disease, enabling conversion to resectability and facilitating contralateral liver regeneration [10].

Advancements in imaging, catheter-based delivery systems, and precision dosimetry continue to refine the application of both techniques. As these modalities evolve, radiation segmentectomy and lobectomy are anticipated to assume a more prominent role within the multidisciplinary framework of liver cancer management, offering a compelling oncologic efficacy with favorable safety profiles.

### 3.4. Outcomes

Radiation segmentectomy has emerged as a compelling alternative to surgical resection, particularly for patients with early-stage HCC who are not candidates for surgery. The efficacy of this technique is underscored by its capacity to achieve robust local tumor control and prolonged survival outcomes. A pivotal retrospective analysis by Lewandowski et al. demonstrated a 2-year local tumor control rate of 90%, with a median overall survival exceeding 30 months for patients treated with segmentectomy [29]. These findings were corroborated by the LEGACY trial, which reported an objective response rate exceeding 90% and a median overall survival of 44.6 months [30]. Additionally, the DOSISPHERE-01 trial underscored the advantages of personalized high-dose Y90 therapy. In this study, patients receiving a high-dose segmentectomy achieved an objective response rate of 71% and a median overall survival of 26.6 months, significantly surpassing the 10.7 months observed in the standard-dosing cohort [16]. Collectively, these data establish radiation segmentectomy as a viable curative strategy for patients who are otherwise ineligible for surgical interventions, while emphasizing the critical role of advanced dosimetry in optimizing therapeutic outcomes.

Radiation lobectomy, in contrast, serves a dual purpose in the management of advanced or unresectable HCC. First, it effectively downstages tumors, enabling previously ineligible patients to become candidates for curative interventions such as surgery or liver transplantation [1]. Second, it induces hypertrophy in the contralateral liver lobe, thereby expanding future treatment options for patients with limited hepatic reserves. A study by Salem et al. demonstrated that 80% of patients undergoing radiation lobectomy achieved a sufficient contralateral hypertrophy within a median of 3 months, facilitating safe surgical resection or transplantation [32].

Comparative studies highlight distinct advantages and limitations between the two approaches. Radiation segmentectomy is associated with superior tumor control in localized disease, particularly when confined to one or two hepatic segments [9,29,36]. It is ideal for patients with a preserved liver function and without vascular invasion [37]. Conversely, radiation lobectomy is preferred when contralateral hypertrophy is required or when multifocal disease limits segmental targeting [31]. In a retrospective matched cohort study, Vouche et al. reported that segmentectomy yielded higher local control rates than lobar approaches, but lobectomy was more likely to facilitate future resection or transplant in borderline surgical candidates [37].

Patient selection in the setting of portal vein thrombosis (PVT) is critical when choosing between radiation segmentectomy and lobectomy. Segmentectomy is generally avoided in cases of main or lobar PVT due to increased ischemic risk and reduced efficacy; however, it may still be considered for patients with segmental or subsegmental PVT if adequate arterial perfusion is preserved [16,35]. In contrast, radiation lobectomy is better suited to patients with ipsilateral PVT, provided the contralateral portal vein remains patent, as it can induce hypertrophy and potentially enable future resection [32].

These findings collectively highlight the complementary roles of radiation segmentectomy and lobectomy within the therapeutic arsenal of TARE. While segmentectomy is particularly suited for localized, early-stage disease, lobectomy offers a robust solution for advanced cases requiring tumor downstaging or contralateral liver regeneration. Both techniques exemplify the versatility of Y90 TARE in addressing the diverse clinical challenges posed by hepatic malignancies.

### 3.5. Safety

The safety profiles for both radiation segmentectomy and lobectomy remain favorable, particularly when patients are meticulously selected based on clinical criteria. RILD, though rare, represents a potentially serious complication, with an incidence consistently reported at less than 5% across most studies [32,35]. The incorporation of advanced personalized dosimetry and high-resolution pre-treatment imaging has been pivotal in mitigating these risks [38]. For instance, the DOSISPHERE-01 trial demonstrated that personalized high-dose Y90 therapy for segmentectomy achieved similar rates of adverse events compared to standard-dose regimens, despite delivering significantly higher radiation doses [16]. Commonly reported side effects, such as fatigue and mild abdominal pain, were transient and effectively managed, underscoring the tolerability of these techniques [32].

These findings strongly support the role of radiation segmentectomy and lobectomy as both safe and effective therapeutic alternatives to surgery. In particular, these modalities offer substantial benefits for patients with early-stage or advanced HCC who are ineligible for traditional surgical approaches due to comorbidities, anatomical constraints, or advanced disease characteristics [16]. By leveraging precision techniques and rigorous procedural protocols, these approaches continue to expand treatment options while maintaining a high safety profile [38].

## 4. Personalized Dosimetry

Personalized dosimetry has fundamentally transformed the landscape of Y90 TARE by enabling a precise calibration of radiation doses tailored to the unique characteristics of individual patients and their tumors. Diverging from the traditional one-size-fits-all approach of standardized dosimetry, personalized methods leverage sophisticated imaging modalities such as quantitative technetium-99 m macroaggregated albumin (Tc-99 m MAA) scintigraphy and PET to devise highly individualized treatment strategies [39]. Evidence from landmark studies, including the LEGACY trial, underscores the efficacy of this approach, demonstrating objective response rates surpassing 90% and a median overall survival of 44.6 months in appropriately selected patients [30]. Through its capacity to optimize tumor targeting, reduce radiation exposure to healthy liver parenchyma, and mitigate complications such as RILD, personalized dosimetry represents a significant leap forward in therapeutic precision and patient outcomes.

### 4.1. Clinical Trial Data on Patients with HCC

Pivotal trials, such as DOSISPHERE-01, have illuminated the advantages of personalized dosimetry in the treatment of HCC. This randomized study revealed that patients receiving personalized high-dose Y90 therapy experienced superior outcomes compared to those on standard dosing protocols [16]. Specifically, the high-dose cohort achieved an objective response rate of 71%, nearly doubling the 36% observed in the standard-dose group [16]. Furthermore, median overall survival was extended markedly to 26.6 months in the personalized dosimetry group, compared to just 10.7 months for standard dosing, underscoring the survival benefits conferred by individualized treatment planning [16]. These findings reinforce the potential of tailored radiation delivery to achieve optimal therapeutic outcomes, even in intermediate and advanced stages of HCC.

### 4.2. Outcomes Across BCLC Stages

Personalized dosimetry has demonstrated efficacy across the Barcelona Clinic Liver Cancer (BCLC) staging system [40]. In early-stage HCC (BCLC-A), this approach facilitates curative-intent radiation segmentectomy, yielding local tumor control rates exceeding 90%, a median overall survival of 44.6 months, and objective response rates comparable to surgical resection [30]. For intermediate (BCLC-B) and advanced-stage HCC (BCLC-C), personalized dosimetry has proven instrumental in addressing high tumor burdens and unilobar disease. DOSISPHERE-01 highlighted its capacity to enable tumor downstaging and extend survival in patients previously deemed unsuitable for curative interventions, exemplifying its broad applicability and therapeutic value [16].

### 4.3. Safety and Adverse Events

Personalized dosimetry not only enhances efficacy but also ensures a robust safety profile. The DOSISPHERE-01 trial reported that severe adverse events, including RILD, occurred in less than 5% of patients, with no significant difference between personalized high-dose and standard-dose cohorts [16]. Common mild side effects such as fatigue (12%) and abdominal discomfort (10%) were transient and manageable, reflecting the careful planning and execution enabled by advanced imaging techniques [16]. These safety outcomes highlight the dual benefit of personalized dosimetry in maximizing therapeutic impacts while minimizing risk.

Despite these advancements, current dosimetric models still face limitations, including variability in microsphere distribution, the limited resolution of imaging modalities, and challenges in accurately predicting dose–response relationships across heterogeneous tumor landscapes [41]. To address these constraints, emerging technologies such as artificial intelligence (AI) and computational modeling are being explored to enhance predictive accuracy and treatment planning [42]. AI-driven platforms can assimilate multimodal imaging data and patient-specific variables to optimize dosimetric parameters, while advanced voxel-based dosimetry algorithms offer a more granular spatial resolution of dose distribution [40]. These innovations hold promise in refining dosimetry beyond current standards and further individualizing therapy.

In redefining the standard of care for Y90 TARE, personalized dosimetry has established itself as an indispensable tool in the management of HCC, offering improved outcomes across all stages while maintaining a favorable safety profile. Its integration into clinical practice continues to advance the paradigm of precision oncology, paving the way for innovative, patient-centered approaches in the treatment of hepatic malignancies.

## 5. Combination Therapies with TARE

Emerging evidence highlights the synergistic interplay between Y90 TARE and immunotherapy, largely mediated by the pro-inflammatory effects induced by TARE [43]. This process enhances tumor antigen presentation and increases immune cell infiltration within the tumor microenvironment, fostering an immunologically permissive milieu that potentiates the efficacy of immune checkpoint inhibitors and other immunomodulatory interventions [44].

### 5.1. Clinical Trials Evaluating Y90 TARE and Immunotherapy Synergy

Checkpoint inhibitors such as nivolumab, pembrolizumab, and atezolizumab remain at the forefront of immunotherapeutic strategies for HCC. These monoclonal antibodies disrupt key immune checkpoints, including PD-1, PD-L1, and CTLA-4, thereby reinvigorating the antitumor immune response [45].

Several clinical investigations have substantiated the efficacy of integrating Y90 TARE with immune checkpoint blockade in advanced HCC. A retrospective study by Villalobos et al. (PMID: 10742675) reported that the combination of Y90 TARE with specific checkpoint inhibitors, including nivolumab (PD-1 inhibitor) and atezolizumab/bevacizumab (PD-L1 inhibitor), significantly prolonged OS and enhanced response rates (Table 2) [46]. Specifically, patients undergoing this combination therapy achieved a median OS of 12.9 for the entire cohort months, with specific overall survivals of 16.4 and 10.7 months for the nivolumab and atezolizumab/bevacizumab groups, respectively (Table 2) [46].

In a phase I/IIa trial evaluating the efficacy of Y90 TARE combined with durvalmab, Lee YB et al. found the combination therapy demonstrated promising efficacy and safety. A total of 23 patients received at least one dose of durvalumab combined with Y90 TARE for advanced, unresectable HCC (Table 2) [47]. The median PFS was 6.9 months (95% CI, 5.4–15.2), and the 18-month OS rate was 58.3% (95%CI, 36.4–75.0) (Table 2) [47]. The durvalumab and Y90 TARE combination was well tolerated, with 47.8% (n = 11) of patients experiencing any-grade treatment-related adverse events and 8.7% (n = 2) of patients experiencing grade 3 treatment-related events (Table 2) [47]. While limited in sample size, this preliminary study shows promising results supporting the efficacy of Y90 TARE combination therapy with durvalumab, warranting further evaluation in large-scale clinical trials.

Additionally, the CA 209–678 study by Tai et al., a phase II trial, evaluated the integration of Y90 TARE with nivolumab in patients with advanced HCC (Table 2) [48]. A total of 36 patients received Y90 TARE followed by nivolumab. The results demonstrated an ORR of 30.6% (95% CI, 16.4–48.1) and a DCR of 61.1%, with a median PFS of 3.6 months and an OS of 16.9 months (Table 2) [48]. These findings reinforce the hypothesis that TARE-mediated immune modulation can enhance checkpoint inhibitor efficacy, particularly in biomarker-selected patient subgroups. However, like the study from Lee et al., the CA209–678 study is limited by a small sample size, highlighting the need for additional large-scale clinical trials to evaluate the efficacy of Y90 TARE combination therapies.

The EMERALD-Y90 (NCT06040099) study will evaluate the efficacy of Y90 TARE combination therapy with durvalumab and bevacizumab in a larger patient population [49]. This phase II, single-arm study is actively enrolling and aims to include approximately 100 patients with unresectable HCC. Eligible patients will receive TARE using Y90 glass microspheres, followed by a single of durvalumab and druvalumab + bevacizumab every three weeks until completion of the study. The primary end point will be PFS, and the secondary end points will be safety, tolerability, objective response rate, and OS [49].

### 5.2. Clinical Safety Considerations

Safety data from clinical trials indicate that Y90 TARE in combination with immunotherapy is generally well tolerated. Lee et al. showed low rates of grade 3 adverse events, and no serious adverse events were reported (Table 2) [47]. Similarly, the CA 209–678 trial identified immune-mediated toxicities, such as dermatologic reactions (10%) and hepatic inflammation (7%), as manageable with standard supportive care (Table 2) [48]. RILD, though a potential complication, was observed in fewer than 5% of cases across studies [48,49], underscoring the importance of precise dosimetric planning and meticulous patient selection.

### 5.3. Implications for Clinical Practice

The integration of Y90 TARE with immunotherapeutic regimens presents a novel and synergistic approach to the management of advanced hepatic malignancies. This synergy is believed to be mediated through several mechanisms, including the pro-inflammatory effects of TARE-induced tumor necrosis, which enhances antigen presentation and promotes immune cell infiltration within the tumor microenvironment [50]. Additionally, radiation may upregulate the expression of immune checkpoint molecules and neoantigens, rendering tumors more susceptible to immune-mediated attack [51].

This therapeutic paradigm is particularly relevant for patients demonstrating suboptimal responses to immunotherapy alone, thereby expanding the scope of treatment within a multidisciplinary oncologic framework. As ongoing clinical trials continue to mature, the role of Y90 TARE as a component of standard-of-care treatment is likely to be further substantiated. Future research should focus on refining patient selection criteria, optimizing combination sequencing, and identifying predictive biomarkers to enhance clinical outcomes.

The combination of Y90 TARE with immune checkpoint inhibitors represents a compelling advancement in the treatment of advanced HCC. Data from clinical trials, including Lee et al. and CA 209–678, highlight significant improvements in tumor response rates and patient survival, while maintaining an acceptable safety profile. As research advances, this combinatory approach is poised to become an integral component of HCC management. Further investigations are necessary to establish standardized treatment guidelines and solidify the clinical benefits of integrating Y90 TARE with immunotherapy.

## 6. Imageable Microspheres

TARE utilizes microspheres loaded with radioactive isotopes, such as Y90, which are administered via the hepatic artery. These microspheres selectively localize within the tumor’s microvasculature due to its preferential arterial supply, thereby delivering localized radiation to malignant tissue [52]. The capability to image and track these microspheres post-administration is of paramount importance in verifying accurate delivery and optimizing therapeutic efficacy [53]. Historically, the imaging of Y90 microspheres has been hindered by the isotope’s pure beta-emission properties, precluding direct visualization using conventional imaging modalities [54]. The advancement in and integration of new imaging capabilities allows for the real-time monitoring of microspheres’ distribution, thereby enhancing precision and enabling immediate procedural modifications when necessary.

Contemporary research in microsphere technology has centered on augmenting their functional properties to enhance the efficacy of transarterial therapies. Jia et al. (2022) [55] explores the development of multifunctional microspheres and nanoparticles tailored for transarterial chemoembolization (TACE), a modality analogous to TARE [4]. This study delineates several key innovations, including the engineering of microspheres capable of the modulated, sustained release of chemotherapeutic agents to maximize localized drug bioavailability while minimizing systemic toxicity. Additionally, the formulation of biocompatible, degradable polymer microspheres reduces the risk of long-term foreign body reactions and associated complications [55]. Another critical advancement is the incorporation of imaging agents within microspheres, enabling real-time tracking and post-procedural assessment, thereby optimizing treatment monitoring and therapeutic planning [55]. These advancements collectively contribute to refining the precision of microsphere-mediated therapies, while simultaneously broadening their therapeutic functionalities.

The integration of imageable microspheres into TARE confers several critical clinical advantages. Pre-procedural imaging facilitates comprehensive vascular mapping, allowing for refined microsphere delivery strategies tailored to individual tumor architectures [56]. Intra-procedural imaging empowers clinicians to dynamically assess the microspheres’ distribution, ensuring accurate localization and mitigating unintended radiation exposure to non-target tissues [57]. Post-procedural imaging provides an objective assessment of microsphere dispersion and radiation dosimetry, guiding subsequent therapeutic decision-making and follow-up interventions [58]. The advancements delineated in Jia et al.’s study, particularly within the domain of TACE, underscore the translational potential of similar innovations within TARE. The advent of multifunctional, imageable microspheres heralds a transformative shift in the landscape of transarterial therapies, fostering enhanced precision, safety, and therapeutic efficacy in the management of HCC.

In a first-in-human trial, imageable Y90 glass microspheres were used to treat six patients with unresectable HCC, demonstrating a 50% complete response rate at six months and no treatment-related adverse events of grade 3 or higher [59]. The study utilized a prospective, single-center design to evaluate the safety, feasibility, and imaging characteristics of these microspheres. Each patient received a single administration of Y90 microspheres via TARE, with imaging assessments conducted using CT and single-photon emission computed tomography (SPECT) to track their distribution and therapeutic impact [59]. Although preliminary, the study data show that TARE with imageable microspheres was safe and effective, and that the microsphere distribution was visible on CT correlated with SPECT imaging, providing valuable CT-based tumor-targeting information and supporting the potential for real-time imaging guidance in future clinical applications [59].

Despite these advancements, several potential drawbacks and limitations of imageable microspheres and TARE must be acknowledged. First, the integration of imaging agents into microspheres can increase production complexity and cost, potentially limiting widespread adoption and accessibility in lower-resource settings [60]. Moreover, regulatory approval processes for novel composite microsphere agents, especially those incorporating imaging and therapeutic components, can be prolonged and complex due to stringent safety and efficacy evaluations [60]. Another limitation is the current resolution of the imaging modalities used to track these microspheres. While SPECT and CT-based imaging allow for visualization [61], their spatial resolution remains inferior to that of diagnostic MRI or PET, which may hinder the precise localization of microspheres in smaller or deeper lesions [62]. Additionally, there remains a risk of misinterpretation due to imaging artifacts or partial volume effects, potentially compromising accurate dosimetry [62]. Lastly, although initial trials suggest safety, the long-term biocompatibility of new imageable microsphere formulations, particularly those incorporating heavy metals or synthetic contrast agents, warrants further study [62].

The continual evolution of microsphere technology, with a strong emphasis on advanced imaging integration and multifunctionality, holds substantial promise in revolutionizing TARE for hepatic malignancies. Ongoing research and clinical trials will be instrumental in the translation of these innovations into routine clinical practice, ultimately advancing the precision and effectiveness of transarterial therapeutic strategies.

## 7. Pressure-Enabled Y90 Delivery Systems

Traditional TARE using Y90 microspheres is often hindered by tumors’ vascular resistance [63], leading to a heterogeneous microsphere distribution and suboptimal radiation deposition within malignant tissues [64]. This limitation compromises therapeutic efficacy by failing to uniformly target tumor regions, thereby reducing treatment response rates. To overcome these challenges, pressure-enabled drug delivery systems (PEDDSs) [65] have been developed as an innovative approach to optimize microsphere penetration and enhance therapeutic outcomes through the strategic modulation of intravascular pressure.

PEDDSs function by transiently altering hemodynamic conditions during the infusion of Y90 microspheres, thereby mitigating intratumoral vascular resistance and facilitating deeper microsphere penetration into the tumor microenvironment [66]. This approach significantly improves microsphere biodistribution and enhances radiation dose homogeneity [67], addressing the limitations of conventional TARE. Particularly, PEDDSs prove advantageous in complex anatomical and hemodynamic scenarios, such as arteriovenous shunting and tortuous vasculature, where standard delivery techniques are insufficient [68]. By enhancing precision in microsphere deployment, PEDDSs minimize radiation exposure to healthy hepatic parenchyma while maximizing tumoricidal effects.

While early, emerging clinical evidence suggests that PEDDSs may confer superior oncologic outcomes compared to conventional Y90 delivery systems. Recently, a study assessing PEDDSs in clinical practice demonstrated that despite patients treated with PEDDSs presenting with a higher baseline disease burden, treatment outcomes were comparable to those achieved with standard delivery methods [68]. The study found that patients undergoing PEDDS procedures had a higher baseline disease burden, with a mean Charlson comorbidity index of 6.5 compared to 5.8 in non-PEDDS patients [68]. Additionally, 33.7% of PEDDS patients had prior clinical complications related to their underlying disease, versus 31.0% in the non-PEDDS group [68]. Despite these complexities, the post-procedure healthcare resource utilization and clinical complications were comparable between PEDDS and non-PEDDS groups [68]. These findings suggest that PEDDS can achieve similar clinical outcomes to standard delivery methods, even in patients with more advanced disease profiles. These findings reinforce the potential of PEDDSs to enhance treatment efficacy and safety, paving the way for further exploration in larger prospective trials.

However, concerns remain regarding the potential for vascular complications associated with pressure-mediated infusion techniques. Elevated infusion pressures, while beneficial for overcoming vascular resistance, may increase the risk of endothelial injury or non-target embolization in sensitive hepatic vasculature [69,70]. Furthermore, the cost of PEDDSs may limit widespread accessibility, particularly in resource-constrained healthcare settings. Compared to conventional catheter-based delivery systems, PEDDSs introduce additional equipment and procedural complexity, which may contribute to higher upfront costs and training requirements [71]. Nonetheless, their potential to improve the dose distribution and achieve similar or superior clinical outcomes in more complex patients suggest a favorable risk–benefit profile that warrants further investigation in prospective randomized trials [69,70,71,72,73].

The future trajectory of PEDDSs in HCC treatment necessitates further clinical validation through large-scale trials and integration with advanced imaging modalities, including real-time intraprocedural imaging and artificial intelligence-driven dosimetry. Such advancements will enable precise, patient-specific treatment planning, ensuring an optimal microsphere distribution tailored to tumors’ morphology and vascular architecture. Additionally, ongoing research is investigating the potential synergy of PEDDSs with emerging systemic therapies, including immune checkpoint inhibitors and molecularly targeted agents, to enhance the therapeutic index [74]. As these innovations continue to evolve, PEDDSs are poised to redefine the landscape of liver cancer intervention, establishing themselves as a critical component of precision oncology in interventional radiology.

## 8. Y90 Surrogates

Y90 surrogates, particularly Ho-166, are emerging as significant alternatives in TARE, owing to their distinct physical and dosimetric properties that cater to specific clinical demands. Ho-166 uniquely combines therapeutic and imaging capabilities, emitting beta radiation for tumor ablation and gamma radiation for imaging compatibility. Its paramagnetic properties enable high-resolution imaging through MRI, a feature that sets it apart from other radionuclides in TARE. These dual functionalities enhance treatment precision by allowing real-time monitoring and post-procedure evaluation.

### 8.1. Current Use in Clinical Practice

Ho-166 radioembolization has been increasingly integrated into clinical practice for treating HCC, offering therapeutic advantages such as a higher biological effective dose due to its shorter half-life [11], leading to a potentially improved tumor response, while minimizing radiation exposure to surrounding tissues. Additionally, Ho-166 enables real-time quantitative SPECT imaging and MRI visibility, which Y-90 lacks, allowing for superior treatment planning, real-time dosimetry, and more precise post-procedure assessment over traditional Y90 therapies [11]. Recent clinical trials have provided valuable insights into its safety, efficacy, and potential role in HCC management.

A study by Smits et al. (2022) evaluated the safety and efficacy of Ho-166 radioembolization in 31 patients with BCLC stage B or C HCC [75]. The findings indicated that common grade 1 or 2 clinical toxicities included fatigue (71%), back pain (55%), and ascites (32%), with less than 10% experiencing grade 3–5 toxicities [75]. At the three-month follow-up, 54% of target lesions demonstrated complete or partial responses according to modified RECIST criteria [75]. The median overall survival was reported at 14.9 months [75], supporting the potential of Ho-166 as a viable treatment option.

Further supporting these findings, a study by Radosa et al. (2019) assessed the feasibility and safety of Ho-166 radioembolization in patients with unresectable HCC. Nine patients with unresectable HCC underwent Ho-166 radioembolization to assess its feasibility and safety [76]. The procedure was technically successful in all cases, with no significant adverse events reported during or after treatment [76]. The study concluded that Ho-166 radioembolization is a feasible and safe treatment option for patients with unresectable HCC [76].

Additionally, the HORA EST HCC trial investigated the use of adjuvant Ho-166 radioembolization following radiofrequency ablation in early-stage HCC patients. This dose-finding study aimed to determine the optimal dosing parameters to maximize therapeutic outcomes while minimizing adverse effects [77]. The trial’s outcomes are anticipated to refine treatment protocols and enhance the efficacy of combination therapies in early-stage HCC management.

Collectively, these studies highlight the growing body of evidence supporting the integration of Ho-166 radioembolization into clinical practice for HCC treatment. The dual therapeutic and imaging capabilities of Ho-166 not only facilitate precise treatment delivery but also enable real-time monitoring, thereby potentially improving patient outcomes.

Despite these promising clinical results, the widespread adoption of Ho-166 radioembolization is currently limited by several practical considerations. As a relatively novel agent, Ho-166 is not yet widely available outside of clinical trial settings, and access may be restricted to specialized centers with appropriate radiopharmacy capabilities and imaging infrastructure [78]. Regulatory approval processes also vary internationally, contributing to inconsistent adoption across institutions. Moreover, the production cost of Ho-166-labeled microspheres is higher than that of Y90 due to complex synthesis and the integration of dual-modality imaging technologies, which may pose economic barriers to broader use [78,79,80].

### 8.2. Future Directions for Ho-166

Ongoing research is focused on refining dosimetric models to maximize the therapeutic efficacy of Ho-166 while minimizing adverse effects. Studies such as the HORA EST HCC trial are actively evaluating optimal dosing regimens for Ho-166 to enhance treatment outcomes in patients with early-stage HCC [77]. Additionally, research is investigating the combination of Ho-166 radioembolization with other locoregional or systemic therapies, such as immune checkpoint inhibitors or tyrosine kinase inhibitors, to improve overall survival rates in patients with advanced liver cancer [75,81]. These combinatorial approaches could expand the use of TARE beyond its current indications, potentially improving long-term disease control.

In parallel, the exploration of next-generation surrogates for Y90 is advancing, with researchers investigating alternative radionuclides such as Rhenium-188 (Re-188) and Lutetium-177 (Lu-177). Re-188, a generator-produced isotope, presents a cost-effective and readily available option, with high-energy beta emissions suitable for hepatic radioembolization [82,83]. Meanwhile, Lu-177, widely used in peptide receptor radionuclide therapy (PRRT), is being studied for its potential in liver-directed treatments due to its favorable radiation characteristics and theranostic applications [82]. These emerging alternatives may offer unique advantages over both Y90 and Ho-166, particularly in scenarios where specific radiation penetration depths or imaging modalities are preferred.

## 9. Future Directions

The future of Y90 chemoembolization resides in its ability to evolve through innovation and collaborative multidisciplinary approaches. Recent clinical trials have illuminated the potential for integrating TARE with systemic immunotherapies such as nivolumab and atezolizumab [33,48,49]. These combinations have demonstrated the capability to significantly improve clinical outcomes in advanced HCC, paving the way for TARE to expand beyond its traditional role as a liver-focused therapy to a systemic treatment modality when paired with immune checkpoint inhibitors.

Parallel advancements in imaging and dosimetry techniques, such as quantitative Tc-99 m macroaggregated albumin (MAA) scintigraphy and Y90 PET-CT, have enhanced the precision of radiation delivery [84]. These innovations reduce toxicities and optimize therapeutic efficacy by enabling tailored, patient-specific treatment plans. Clinical studies like the LEGACY trial underscore how these advancements have led to superior outcomes, particularly in early- and intermediate-stage HCC [30], through improved tumor targeting and personalized treatment strategies.

Personalized dosimetry in Y90 TARE for HCC treatment will increasingly leverage these advanced imaging technologies to refine dose optimization further. The ability to assess the heterogeneous distribution of radiation within the tumor and normal liver tissue offers the potential for individualized dose prescriptions that account for tumor size, shape, vascularization, and other biological factors. The real-time monitoring of radiation delivery will enable clinicians to adjust treatment plans dynamically, improving the safety and effectiveness of TARE. Moreover, personalized dosimetry could allow for the customization of Y90 microsphere delivery to minimize radiation exposure to healthy tissue, thus reducing side effects and enhancing patient outcomes.

Moreover, expanding the applications of TARE to other malignancies, such as metastatic colorectal cancer (mCRC) and intrahepatic cholangiocarcinoma, remains an active area of investigation [85]. Emerging data suggest [86] that these cancers could benefit from innovative approaches that integrate radiobiological principles and advances in microsphere engineering, offering the potential for broader applicability.

Innovations in microsphere technology, including the development of imageable and biodegradable microspheres, have further increased the versatility of TARE [55]. These advancements enhance real-time treatment monitoring and expand safety margins. Additionally, the exploration of alternative radionuclides such as Ho-166 provides complementary options to Y90, broadening the therapeutic landscape and addressing diverse clinical challenges.

Despite these promising developments, several barriers may hinder widespread implementation. Challenges such as reimbursement constraints [87,88], lack of standardization across institutions, and the need for enhanced physician training must be addressed to facilitate broader adoption [89]. Furthermore, the integration of artificial intelligence into personalized treatment planning offers the exciting potential to optimize dosimetry and improve clinical decision-making, but its incorporation into clinical workflows remains in the early stages and requires further validation [90,91].

Collectively, these innovations reinforce TARE as a cornerstone of personalized oncology. By addressing complex challenges in cancer management, improving therapeutic precision, and expanding its indications, Y90 TARE continues to shape the future of interventional oncology with a focus on improving patient outcomes and quality of life.

## 10. Conclusions

In conclusion, Y90 TARE has emerged as a transformative and highly effective treatment modality for HCC and metastatic liver disease. Through its targeted delivery of radioactive microspheres, TARE offers robust tumor control with minimized toxicity, providing a valuable alternative to traditional treatment approaches. The integration of advanced imaging and dosimetry techniques has enhanced the precision and safety of this treatment, enabling personalized therapy tailored to individual patient needs.

The ongoing development of new technologies, such as imageable microspheres, pressure-enabled delivery systems, and novel radionuclides like Ho-166, further strengthens the clinical utility of TARE. These innovations promise to expand the scope of TARE in managing a broader range of hepatic malignancies and improve treatment outcomes by enhancing tumor targeting and minimizing off-target effects. Moreover, the potential synergy between TARE and immunotherapy opens new avenues for treating advanced liver cancers, offering hope for patients with limited treatment options. While these advancements are promising, their widespread clinical implementation will depend on several factors, including regulatory approvals, manufacturing scalability, and integration into existing treatment protocols.

As TARE continues to evolve, its role as a foundation of HCC treatment is solidified. The combination of precision therapy, improved safety profiles, and expanding indications positions Y90 radioembolization as a key player in the multidisciplinary management of liver cancer. Moving forward, ongoing research and clinical trials will likely refine its applications, further establishing TARE as an indispensable tool of interventional oncology, ultimately improving patient outcomes and quality of life.

## Figures and Tables

**Table 1:** Selection criterion for Y90 TARE treatment in HCC.

Selection Criterion	Details
Liver Function	Preserved hepatic function, typically characterized by Child–Pugh class A or early B, without significant portal hypertension or ascites [20].
Tumor Burden	Predominantly liver-dominant disease with minimal extrahepatic dissemination and no significant biliary obstruction [9].
Vascular Anatomy	Favorable hepatic arterial anatomy to enable precise tumor targeting and mitigate the risk of non-target radiation exposure.
Performance Status	ECOG performance status of 0–2 [7].
Contraindications	Uncorrectable arteriovenous shunting, substantial pulmonary shunting, or severe renal or hepatic insufficiency [10].

**Table 2:** Clinical trials evaluating combinations of Y90 TARE and systemic therapy to treat HCC. HCC: hepatocellular carcinoma. Y90 TARE: Yttrium-90 transarterial radioembolization. OS: overall survival. PFS: progression-free survival. ORR: overall response rate. DCR: disease control rate. AFP: apha-fetoprotein. RILD: radiation-induced liver disease.

	Villalobos et al. [46]	Lee YB et al. [47]	Tai et al. [48]
Patient Population	Advanced HCC patients receiving Y90 TARE with immune checkpoint inhibitors (nivolumab, atezolizumab/bevacizumab).	Advanced, unresectable HCC (Child–Pugh < 7, BCLC stage B or C).	Advanced HCC patients receiving Y90 TARE with nivolumab.
Study Comparisons	Y90 TARE + immunotherapy vs. immunotherapy alone.	Y90 TARE + durvalumab.	Y90 TARE + nivolumab vs. historical control data.
Key Findings	Median OS: 12.9 months for the entire cohort, 16.4 months for nivolumab, and 10.7 months for atezolizumab/bevacizumab).	Median PFS: 6.9 months (95% CI, 5.4–15.2). ORR: 83.3% (95% CI, 62.6–95.3).	ORR: 30.6% (95% CI, 16.4–48.1), DCR: 61.1%, median PFS: 3.6 months, OS: 16.9 months. Supports TARE-mediated immune modulation.
Safety Data	Well tolerated; main AEs included fatigue, hepatic transaminase elevations, and GI disturbances. Grade 3 + toxicities < 10%.	47.8% (n = 11) experienced any-grade treatment-related events. 8.7% (n = 2) experienced grade 3 treatment-related adverse events (neutropenia and fever). No serious events reported.	Immune-mediated toxicities: dermatologic reactions (10%), hepatic inflammation (7%). RILD in <5%.
